# Allometry of bud dynamic pattern and linkage between bud traits and ecological stoichiometry of *Nitraria tangutorum* under fertilizer addition

**DOI:** 10.7717/peerj.14934

**Published:** 2023-03-21

**Authors:** Qinghe Li, Na Duan, Chenggong Liu, Huiqing Li, Lan Xu

**Affiliations:** 1 Research Institute of Forestry, Chinese Academy of Forestry, Beijing, China; 2 Key Laboratory of Tree Breeding and Cultivation, National Forestry and Grassland Administration, Beijing, China; 3 Experimental Center of Desert Forestry, Chinese Academy of Forestry, Dengkou, Inner Mongolia, China; 4 Department of Natural Resource Management, South Dakota State University, Brookings, SD, United States of America

**Keywords:** *Nitraria tangutorum*, Aboveground bud, Fertilizer addition, Ecological stoichiometry, Allometry, Bud dynamic pattern, Bud fate

## Abstract

Affected by the pressure and constraints of available resources, plant growth and development, as well as plant life history strategies, usually vary with environmental conditions. Plant buds play a crucial role in the life history of woody plants. *Nitraria tangutorum* is a common dominant woody species in desertified areas of northern China and its growth is critical to the desert ecosystem. Revealing the allometry of *N. tangutorum* aboveground bud fates and the linkage between bud traits and plant nutrient contents and stoichiometric ratios can be useful in understanding plant adaptation strategy. We applied seven nitrogen and phosphorus fertilizer addition treatments to natural *N. tangutorum* ramets in Ulan Buh Desert in three consecutive years. We surveyed three types of aboveground buds (dormant buds, vegetative buds, and reproductive buds) in each *N. tangutorum* ramet, then measured the plant carbon (C), nitrogen (N), and phosphorus (P) contents and ratios during three consecutive years. We specified that reserve growth potential (RGP), vegetative growth intensity (VGI) and sexual reproduction effort (SRE) are the three indices of bud dynamic pattern. The results showed that the bud dynamic pattern of *N. tangutorum* ramets differed significantly among different fertilizer addition treatments and sampling years. The allometry of RGP, VGI, and SRE was obvious, showing size dependence. The allometric growth relationship fluctuated among the sampling years. The linkage between bud traits and plant stoichiometric characteristics of *N. tangutorum* ramets showed close correlation with plant P content, C:P and N:P ratios, no significant correlation with plant C content, N content and C:N ratio. These results contribute to an improved understanding of the adaptive strategies of woody plants growing in desert ecosystems and provide insights for adoption of effective measures to restore and conserve plant communities in arid and semi-arid regions.

## Introduction

Growth and reproduction are among the most basic life-history functions in plants. The total resources available to plants at a given time is often limited (She et al., 2017). Therefore, only by strategically allocating available resources to their constituent organs (stems, leaves, roots, and reproductive structures) can plants optimize their growth and survival (Yu et al., 2020). Under natural selection pressures and constraints, plants often modify their resource allocation patterns to maximize their fitness and adaptability (Bonser & Aarssen, 1996; Weiner, 2004; She et al., 2017). For instance, the cost of reproductive structures for plants growing in a harsh environment increases as an individual plant grows larger (Guo et al., 2012). Alternatively, resource allocation may be dedicated to vegetative growth, reproduction, or remaining dormant depending on the fate of the meristems (Weiner et al., 2009; Lundgren & Des Marais, 2020).

The trade-off between vegetative and reproductive growth is a basic strategy of plants for population maintenance and survival, and is species-specific (Fournier et al., 2020). Typically, a vascular plant sporophyte is organized into an aboveground organ system, including stems and leaves, and a belowground organ system, comprising roots and vegetative reproductive structures such as rhizomes. The aboveground shoot system of a plant, resulting from the development and outgrowth of aboveground meristems (*i.e.,* buds), is an embodiment of plant morphological structure for photosynthesis and other functions, such as sexual reproduction. The aboveground shoot architecture is the product derived from the development of aboveground buds of plants (Klimešová et al., 2019). However, not all buds of a plant are identical in terms of size, morphological and physiological characteristics, function, and behavior; different bud types carry different costs and confer different benefits to the plant (Vesk & Westoby, 2004).

The aboveground buds of a perennial plant during the growing season can be classified into three types according to the ultimate bud developmental fate: dormant buds, which are mainly reserve growth points and regenerate after disturbance, or help the plant to survive adverse environmental conditions (Vayssières et al., 2020); vegetative buds give rise to leaves or stems, enabling spatial growth of the shoots and ensuring the formation of new photosynthetic organs to supply carbohydrates to non-photosynthetic structures of the plant (Samach & Smith, 2013); and reproductive buds develop into sexual reproductive structures, such as flowers and inflorescences. Together, the three types of buds determine the plant’s aboveground morphological structure. Additionally, the number of phytomers and the organization of bud fate have been useful for characterizing aboveground morphology and structure in numerous plant species (Prats-Llinàs et al., 2019). The number and type of buds determine carbon (C) allocation patterns and yield (Kaur et al., 2012), which are likely associated with species allometry, and then bud phenology alters the number of reproductive units (Fournier et al., 2020). Allometry and resource allocation has been reported in some plant populations, with the focus on the allometric growth of plant size measured by biomass (Palacio, Millard & Montserrat-Marti, 2006; Schafer & Mack, 2014).

However, the use of biomass in allocation measures has limitations in that it does not determine plant form. Moreover, differences in plant fitness can be, in part, a consequence of differences in allocation pattern to meristems (*i.e.*, reproductive, vegetative, and dormant meristems); in this case, the adaptive strategy of meristem allocation instead of biomass allocation might be more practical (Bonser & Aarssen, 1996). Based on a meristem allocation model, the life-history attributes of flowering plants can be defined as the number of meristems associated with a given function relative to the total number available for that assignment, *i.e.,* sexual reproductive effort can be measured as the ratio of reproductive *versus* (vegetative + dormant) meristems, growth intensity can be measured as the ratio of vegetative meristems *versus* (dormant + reproductive) meristems, and apical dominance can be measured as the ratio of dormant meristems *versus* (vegetative + reproductive) meristems (Bonser & Aarssen, 1996; Finley & Aarssen, 2022). The size of potential bud banks enables plants to overcome meristem limitation from severe damage (*e.g.*, from herbivory, fire, or drought events) (Klimešová et al., 2014) and to optimize tiller recruitment under environmental variation (Russell et al., 2017). Three indices of bud dynamic pattern, expressed as reserve growth potential (RGP), vegetative growth intensity (VGI), and sexual reproduction effort (SRE), can be calculated from the number of each bud type.

Some plant traits show consistent patterns of size dependence. Among the many factors affecting plant reproductive allocation and reproductive output, size dependence has become the focus of research exploring the theory of plant life history (McIntosh, 2002; Niklas, 2005). From an allometric perspective, allocation is considered as a size-dependent process: allometry is the quantitative relationship between growth and allocation (Weiner, 2004). However, when plant size has been measured by the number of buds, it has been more done to explore the size of the belowground bud bank for vegetative reproduction. To date, plant size measured by aboveground buds has been relatively unexplored, and even less research has been conducted on strategies for allocation of available plant resources among different types of buds in a harsh habitat (*e.g.*, on xerophytes and desert plants). The dynamic of aboveground buds indicates the change in morphological structure of the aboveground shoot system. On the one hand, management practices impact process-based allometry of the aboveground architecture of trees (Brym & Ernest, 2018), and on the other hand, the aboveground buds are affected by environmental resource availability. Therefore, the allometry laws of the aboveground bud dynamic pattern may reflect the adaptive life-history strategies of plants in a certain environment.

In recent years, ecological stoichiometry has played an important role in analysis of the composition, structure, and function of a community and ecosystem, especially C, nitrogen (N), and phosphorus (P), and their plant tissue contents and ratios can be used as indicators of ecosystem nutrient limitation (Chen & Chen, 2021). For plants, the variation in chemical traits is a result of a trade-off between stoichiometric homeostasis and plasticity (Sistla & Schimel, 2012), which are under the control of biological and environmental factors (Zhao et al., 2018). Previous studies of ecosystem stoichiometry have suggested that the terrestrial ecosystems biosphere may undergo a shift from N limitation to a broader P limitation, or N and P co-limitation in the future (Peñuelas et al., 2012). Therefore, with increased variation in nutrient heterogeneity, it is reasonable to assume that plant growth is affected by N or P enrichment (Elser et al., 2007; Yuan et al., 2019). For example, fertilization can cause changes in carbohydrate metabolism, transport, and accumulation in buds (Zhou et al., 2020), and also can promote bud differentiation and flowering but only when a proper amount of nutrient is applied (Sønsteby, Solhaug & Heide, 2016). Thus, it is critical to study plant form and ecological stoichiometry traits under continuously varying nutritional environments. However, there remains a lack of systematic recognition of bud developmental fates, especially in the allometry of bud dynamic pattern under fertilization and/or nutrient addition.

**Figure 1:**
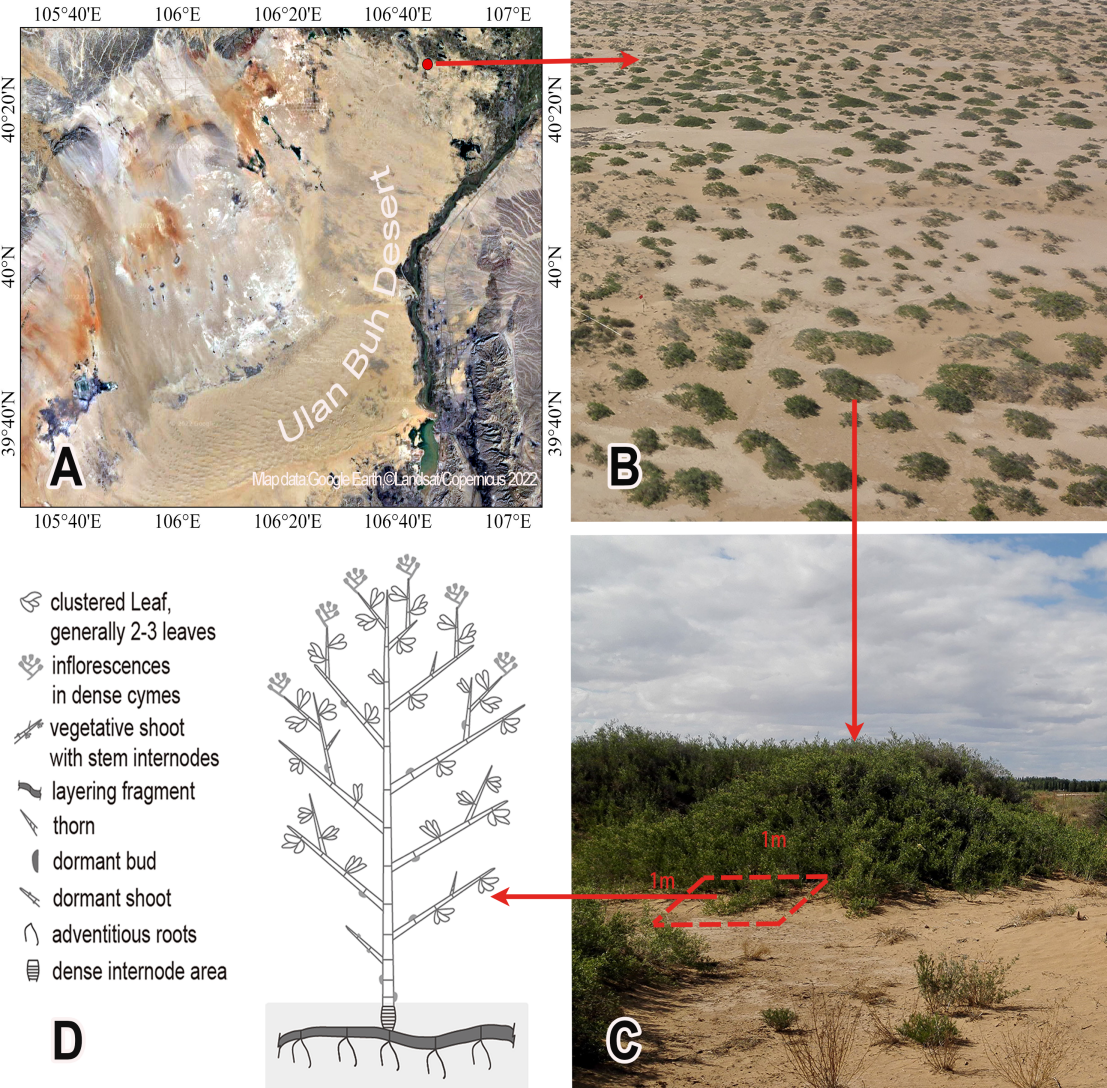
*Nitraria tangutorum* nebkhas vegetation landscape (B) and the established quadrats (C) in the study site (A) and one *N. tangutorum* ramet morphology (D). (A) The location of the study site (Map data: Google Earth, ©Landsat/Copernicus, 2022); (B) *Nitraria tangutorum* nebkhas vegetation landscape in the study site (Photo credit: Qinghe Li); (C) One nebkha (Photo credit: Qinghe Li) and the established quadrats along the margin of one *N. tangutorum* nebkha; (D) The sketch diagram of one *N. tangutorum* ramet morphology.

*Nitraria tangutorum* Bobrov, a native woody species to the arid and semi-arid areas of northwest China, is a small, typical desert clonal shrub and a dominant species of desert plant communities. As an important component of the desert ecosystem, *N. tangutorum* not only has a well-developed root system, but also sexual and asexual reproductive characteristics, such as clonal reproduction by layering to form nebkhas of various sizes (Fig. 1B). This species plays a critical role in maintaining fragile ecosystems in desert regions by stabilizing moving sand dunes, reducing wind speed, and preventing desertification (Li et al., 2013; Liu et al., 2022). In the growing season, the aboveground buds of a ramet also play a crucial role in clonal propagation by layering. To examine how exogenous addition of N and P influence meristem growth in perennials, especially in desert plants, we selected *N. tangutorum* in Ulan Buh Desert as the study subject. We investigated how the effect of nutrient addition on the growth of desert plants is influenced by the bud dynamics, and how the plant C, N, and P ratios indicate changes in plant buds, especially the linkage with plant bud pattern and its plasticity. Our primary goals were as follows: (i) to reveal the allometric differences in *N. tangutorum* bud patterns in response to different N and P addition treatments; (ii) to examine whether P addition is a more important driver of change in bud dynamics of *N. tangutorum* than N addition, and whether plant P content and P-related ratios are more strongly correlated with bud traits of *N. tangutorum* ramets; and (iii) to explore the plasticity of bud dynamics in *N. tangutorum* and the relationship with plant C, N, and P contents and ecological stoichiometric ratios by performing a multi-gradient fertilizer-addition field experiment.

## Material and Methods

### Site description

The study site was located in western Inner Mongolia, in the northeastern region of the Ulan Buh Desert, a temperate arid desert in central North China (40.31°–40.43°N, 106.41°–106.75°E) (Fig. 1A). The climate is characterized by a temperate continental monsoon with prevailing westerly and northwesterly winds, which cause major wind-driven transport of sand sediments. The annual average wind speed is 3.7 m s^−1^. The mean annual precipitation is 145 mm and evaporation is 2,381 mm. The mean annual temperature is 7.6 °C, the absolute maximum temperature is 38.2 °C, and the minimum temperature is −34.2 °C. The frost-free season is 136–205 days. The soil comprises river-deposited clay and scattered sandy soil stabilized by *N. tangutorum* nebkhas (Fig. 1B). The *N. tangutorum* nebkhas plant community in the inter-dune areas is dominated by *Artemisia ordosica* Krasch., *Artemisia sphaerocephala* Krasch., *Agriophyllum squarrosum* (L.) Moq., *Salsola beticolor* Iljin, *Corispermum mongolicum* Iljin, and *Psammochloa villosa* (Trin.) Bor (Li et al., 2013). There are rarely other species growing within the clonal ramet populations of *N. tangutorum* nebkha.

### Experimental and treatment design

The experiment for this study was replicated across three years (2015 to 2017) in one location. In mid-September 2014, a natural *N. tangutorum* nebkhas area (3–5 km^2^) was selected, as a typical representative of the *N. tangutorum* nebkhas community, with an average nebkha size of approximately 20 ±4 m^2^. Within the selected area, 63 permanent quadrats (1 m ×1 m) were established along the margin of randomly selected *N. tangutorum* nebkhas having similar ramet size and developmental stage (Fig. 1C). Within each quadrat, all *N. tangutorum* layered stems of each ramet were severed to ensure that the ramet was independent. Within each quadrat, one ramet with almost identical bud traits and branch numbers was selected and permanently marked for bud sampling in this study.

Based on the soil N and P contents in the experimental field, seven N and P fertilizer addition treatments were designed and randomly assigned to individual quadrats with nine replicates per treatment. The seven treatments comprised three levels of N fertilizer (0, 10, and 20 g m^−2^ of 47% urea (Huashan^®^, Shanxi, China)) mixed with 3 g m^−2^ calcium superphosphate fertilizer (Gezhouba^®^, Yichang, China), three levels of P fertilizer (0, 6, and 12 g m^−2^ of calcium superphosphate with 12% available P_2_O_5_) mixed with 3 g m^−2^ urea fertilizer, and a control (no fertilizer addition). These treatments were designated N0, N10, N20, P0, P6, P12, and CK, respectively. The fertilizer treatments were applied to each designated quadrat in early May of each year from 2015 to 2017. Before application, the urea and calcium superphosphate fertilizers were ground into powder, spread evenly in the quadrats, and covered by a small amount of sand.

### Data collection

During the annual flowering period in mid-May from 2015 to 2017, we recorded the numbers of dormant buds (*D*), vegetative buds (*V*), and reproductive buds (*R*) in the selected ramet of each quadrat. In addition to dormant buds between the stem internodes, dormant buds also include thorns and dormant shoots that are inactive in the current year. The vegetative buds include all vegetative shoots and leaf clusters, and the reproductive buds refer to the formed inflorescences. Figure 1D shows a sketch diagram for a ramet morphology.

We first classified the hierarchical level of the stems. The first-order stem was the primary stem emergent from the soil, and the second-order stem arose from the first-order stem. We observed that the stems of the aboveground part of the *N. tangutorum* ramet comprised up to the seventh order. We recorded the number of buds at each stem hierarchical level. The number of each type of bud on the first-order stem was respectively labeled as *D* 1, *V* 1, *R* 1, and so on. We summarized the number of each bud type at each stem hierarchical level to conduct the data analysis.

After recording the number of buds in 2017, we measured the height and base diameter of each ramet using steel tape (HQ-012, Liangjin, Zhengzhou, China) and vernier calipers (0-200, Nscing Es, Nanjing, China), respectively. The ramets were then harvested and transported to the laboratory. The WinFOLIA leaf area analyzer (WinFolia Pro 2016, Régent Instruments Inc., Québec, Canada) was used to scan the leaf blades and calculate the total leaf area. Roots, stems, leaves, fruits, and layered stems were bagged separately and dried in an air-drying oven at 65 °C for 48 h. After drying, the samples were weighed, then ground and sieved, and used for the determination of C, N, and P contents. A CHNOS elemental analyzer (Vario EL III, Elementar Analysensysteme GmbH, Bremen, Germany) was used to analyze the total C and N contents. A UV-Visible spectrophotometer (UV-2550, Shimadzu, Kyoto, Japan) was used to determine the total P content.

### Data analysis

The data for the three types of buds from each ramet across the seven treatments in three consecutive years were used to analyze the importance of the allometry and plasticity in bud dynamic pattern at the flowering period, and the relationship with the ecological stoichiometric traits. We constructed the three following indices for sexual reproduction effort (SRE), reserve growth potential (RGP), and vegetative growth intensity (VGI) to reflect the bud dynamic pattern:

(1) \begin{eqnarray*}\mathrm{SRE}& =R/(V+D)\end{eqnarray*}

(2) \begin{eqnarray*}\mathrm{RGP}& =D/(R+V)\end{eqnarray*}

(3) \begin{eqnarray*}\mathrm{V GI}& =\mathrm{V }/(D+R)\end{eqnarray*}

where *R*, *D*, and *V* are the numbers of reproductive buds, dormant buds, and vegetative buds, respectively, in a *N. tangutorum* ramet.

The regression analysis between the numerator and the denominator for each of the above equations can respectively examined an allometric relationship between the three indices and ramet size. Total number of two types of buds was used as a measure of plant size rather than total number of buds (*R* + *D* + *V*) because the latter can result in spurious correlations that may obscure the relationship.

The data from three consecutive years were averaged to analyze the correlation of the C, N, and P contents and ratios for the whole plant.

All data analyses and figure preparation were performed using R version 4.2.2 (R Core Team, 2022). The ‘vegan’ R packages were used for the PerMANOVA analyses. PAST 3.0 was used to conduct pairwise comparisons after a PerMANOVA analysis. Statistical significance was determined at the 5% significance level (*P* ≤ 0.05). The three indices were log_10_-transformed before conducting standardised major axis (SMA) regression to analyze the allometric relationship with the ‘smatr’ R package. The significance of the difference between 1 and the SMA regression slope was analyzed. A correlation analysis was performed to analyze the relationship between the *N. tangutorum* bud dynamic pattern and ecological stoichiometry. The ‘ggplot2’ R packages were used for graph generation.

## Results

### Response of bud dynamic pattern to fertilizer addition and allometry

The bud dynamic pattern of *N. tangutorum* ramets at the flowering period differed significantly for the main effects (fertilizer addition treatment and sampling year) and the interaction (fertilizer treatment × sampling year) (Table 1). Fertilizer addition changes the three bud numbers of ramets, resulting in different size of ramets. The three bud dynamic pattern traits differed significantly among the three consecutive sampling years. Pairwise comparisons of the effect of the treatment on the *N. tangutorum* ramets showed that the N10, N20, and P0 fertilizer addition treatments all differed significantly from the CK and P6 treatment (Table 2).

**Table 1:** Result of PERMANOVA for the bud dynamic pattern traits in *Nitraria tangutorum*.

	Df	SS	MS	*F* value	R^2^	*P* value	Significance codes
Fertilizer treatment	6	90.22	15.037	3.008	0.06632	0.0075	^**^
Sampling Year	2	237.07	118.536	23.712	0.17427	0.001	^***^
Fertilizer treatment × Sampling Year	12	193.28	16.106	3.222	0.14207	0.001	^***^
Residuals	168	839.82	4.999		0.61734		
Total	188	1360.39			1.00000		

**Notes.**

Df Degree of freedom SS Sums of Squares MS Mean Squares R^2^ the proportion of Sum of Squares from the Total. Sum of Squares

PERMANOVA was based on the Euclidean distance measure.

*P* values were obtained using 9999 permutations of residuals under the full model.

The Significance Codes: 0 ‘***’ 0.001 ‘**’ 0.01 ‘*’.

**Table 2:** The pairwise comparison of bud dynamic pattern traits on the different fertilizer treatment and sampling year.

Fertilizer treatment	N0	N10	N20	CK	P0	P6
N10	1.000					
N20	1.000	1.000				
CK	1.000	0.777	0.945			
P0	1.000	1.000	1.000	0.798		
P6	1.000	0.273	0.105	1.000	0.189	
P12	1.000	1.000	1.000	1.000	1.000	1.000
Sampling Year	2015			2016		
2016	0.003			
2017	0.003			0.009		

**Notes.**

Fertilizer treatment includes seven treatments: N0, 0 g m^−2^ of 47% urea mixed with 3 g m^−2^ calcium superphosphate fertilizer; N10, 10 g m^−2^ of 47% urea mixed with 3 g m^−2^ calcium superphosphate fertilizer; N20, 20 g m^−2^ of 47% urea mixed with 3 g m^−2^ calcium superphosphate fertilizer; CK, no fertilizer addition; P0, 0 g m^−2^ of calcium superphosphate mixed with 3 g m^−2^ urea fertilizer; P6, 6 g m^−2^ of calcium superphosphate mixed with 3 g m^−2^ urea fertilizer; P12, 12 g m^−2^ of calcium superphosphate mixed with 3 g m^−2^ urea fertilizer.

Sampling Year includes three consecutive years: 2015, 2016, and 2017.

Bonferroni-adjusted *P* value was shown in the table.

**Figure 2:**
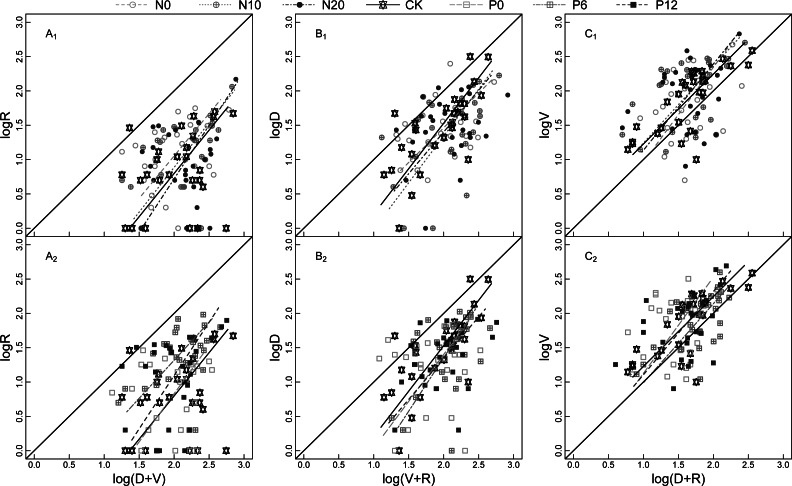
Model II regressions of the allometric relationship for SRE (A_1_ and A_2_), RGP (B_1_ and B_2_), VGI (C_1_ and C_2_) for *Nitraria tangutorum* ramets at flowering stage under fertilizer addition. SRE is sexual reproduction effort; RGP is reserve growth potential; VGI is vegetative growth intensity. R, D, and V are the numbers of reproductive buds, dormant buds, and vegetative buds of *N. tangutorum* ramet respectively. N0, N10, N20, CK, P0, P6, and P12 are seven fertilizer addition treatments, which respectively 0, 10, and 20 g m^−2^ of 47% urea mixed with 3 g m^−2^ calcium superphosphate fertilizer, no fertilizer addition, and 0, 6, and 12 g m^−2^ of calcium superphosphate mixed with 3 g m^−2^ 47% urea fertilizer. A_1_, B_1_, and C_1_ include N0, N10, N20, and CK of fertilization treatments; A_2_, B_2_, and C_2_ include CK, P0, P6, and P12 of fertilization treatments; The leading diagonal solid line represents the slope (equal to 1) of an isometric relationship.

Under the N and P fertilizer addition treatments, the larger the individual ramets of *N. tangutorum*, the greater the RGP, VGI, and SRE values, and the slope of the allometric relationship for the bud dynamic pattern of *N. tangutorum* ramets under the N and P fertilizer addition treatments was significantly greater than 1 (Fig. 2). The RGP of *N. tangutorum* ramets under N10 and P6 treatments (Figs. 2B_1_ and 2B_2_) showed the allometric relationships with the highest slope compared with those of the control treatment (CK). The VGI under the N0 and P0 treatments (Figs. 2C_1_ and 2C_2_) showed the allometric relationships with the highest slope. The allometric relationship with the highest slope for SRE was observed for the N20 treatment followed by P12 (Figs. 2A_1_ and 2A_2_).

**Figure 3:**
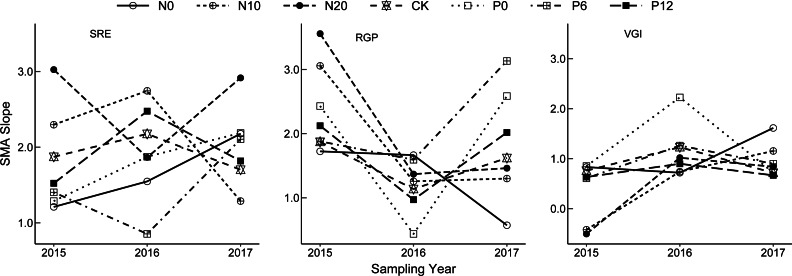
Interannual change of allometric index of bud dynamic pattern in *Nitraria tangutorum*. SRE is Sexual Reproduction Effort; RGP is Reserve Growth Potential; VGI is Vegetative growth intensity. SMA is Standardized Major Axis.

The allometric growth relationship for SRE, RGP, and VGI fluctuated among the sampling years (Fig. 3). Plasticity of bud dynamic pattern generally alternates at flowering developmental stage between different consecutive years. The allometric growth slope of RGP showed a V-shaped change from 2015 to 2017, that of VGI and SRE increased from 2015 to 2016 and declined in 2017. However, the opposite trends were observed for VGI under the N0 treatment, RGP under the N0 treatment, and SRE under the N20 and P6 treatments. This indicates that the strength of allometry and plasticity at flowering stage *N. tangutorum* ramets varies in different years.

### Relationship between bud traits and C, N, and P contents and ratios

We analyzed the correlations between the three bud traits of *N. tangutorum* with the C, N, and P contents and their stoichiometric ratios. The analysis revealed that the total number of all three types of buds or the total number of buds committed to any two fates was significantly negatively correlated with the C:P and N:P ratios, and significantly positively correlated (except for the total number of *D* plus *R* buds) with P content. No significant correlation was observed between any bud trait and the C and N contents and C:N ratio (Table 3). Among the three elements, only the P content and the ratios associated with P showed a significant correlation with the bud trait indices. These results indicated that P was the most important nutrient for bud fate development in *N. tangutorum* ramets.

**Table 3:** Spearman correlation coefficients between bud traits and ecological stoichiometry traits of *Nitraria tangutorum*.

	C(%)	N(%)	P(%)	C:N	C:P	N:P
log(V+R)	0.117	−0.03	0.315^*^	0.117	−0.43^***^	−0.449^***^
log(D+R)	−0.043	0.091	0.231	−0.049	−0.357^**^	−0.29^*^
log(D+V)	0.069	−0.024	0.28^*^	0.1	−0.381^**^	−0.394^***^
log(V+R+D)	0.058	0.004	0.314^*^	0.07	−0.426^***^	−0.42^***^

**Notes.**

R, D, and V are the numbers of reproductive buds, dormant buds, and vegetative buds of *N. tangutorum* ramet respectively; C, N, and P are plant total carbon, total nitrogen, and total phosphorus content respectively.

“*”, “**” and “***” meant correlation coefficients are significant at 0.05, 0.01, and 0.001 levels respectively.

**Figure 4:**
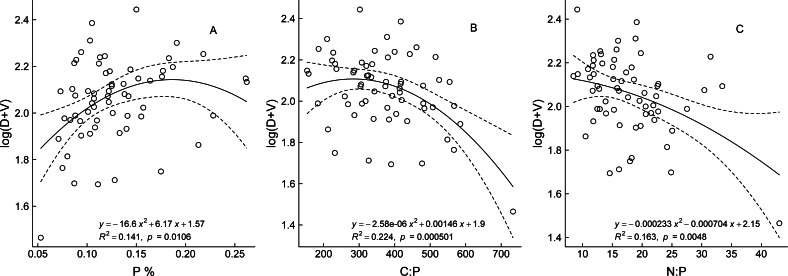
The relationship between bud trait and ecological stoichiometry traits of *Nitraria tangutorum*. D and V are the numbers of dormant buds and vegetative buds of *N. tangutorum* ramet respectively; C, N, and P are plant total carbon, total nitrogen, and total phosphorus content respectively.

The optimal fitted curves between the bud traits and three aforementioned stoichiometric traits showed a quadratic polynomial relationship (Fig. 4). Regression analysis of P content and bud traits of the ramets showed that, with increase in P content, the numbers of all buds on the ramets gradually increased in the early stage, resulting in larger ramets, but when the P content was greater than 0.184%, the number of buds did not increase and did not exhibit the characteristics of larger ramets. The bud numbers trend to decrease slowly with increase in the C:P and N:P ratio values, which showed that the ramet size changed from larger to smaller ramets.

## Discussion

The type and developmental stage of buds greatly influence the morphological plasticity and reproductive efficiency of plants in responses to environmental changes (Silvestro et al., 2020). The buds’ dynamic pattern is an adaptive strategy that responds to a heterogeneous environment by adjusting the relative proportions of the different types of buds to enable the plant to adapt to environmental variability (Bonser & Aarssen, 1996). Usually, the meristems of vegetative buds (*e.g.*, terminal buds and axillary buds) are more nutrient-rich but are more vulnerable to damage (Wang & Dang, 2022). Dormant buds allow plants to protect meristems from unfavorable environmental conditions and to adapt to heterogeneous environments. However, resource allocation within plants is allometric in a broad sense (Weiner, 2004). For example, alterations in the N:P ratio may not only change plant resource allocation to vegetative or reproductive organs, but also may affect the plant growth rate (Zhang et al., 2018). In the present study, the allometry of three bud pattern traits of *N. tangutorum* differed significantly under different N and P addition treatments. The VGI and SRE indices showed more pronounced allometry under the minimum and maximum N and P addition treatments, respectively, which indicated the adaptive response of the bud dynamic in *N. tangutorum* ramets to the differences in resource availability.

The study of the allometric growth law begins with biomass allocation (Watson, 1984). Its main purpose is to summarize the relationship between two variables and usually expressed as a power function (Enquist & Niklas, 2002; Warton et al., 2006; Xu et al., 2022). This relationship is widely present in plants and the analysis of slope or intercept can quantitatively describe the relationship (Niklas & Enquist, 2001; Weiner, 2004). However, a similar approach is difficult to distinguish differences between plants with the same biomass and different plant architecture. Considering this difficulty, the number of meristems has been validated as a surrogate for estimation of biomass allocation (She et al., 2017). Furthermore, the optimal pattern of meristem allocation may change with environmental conditions and the size or number of reproductive units in the terms of plant survival strategies (Bonser & Aarssen, 1996); for example, a larger number of buds provides the plant with increased flexibility for meristem deployment (Kleiman & Aarssen, 2007). In the present study, the three bud dynamic pattern traits of *N. tangutorum* ramets showed the same allometric relationships under the N and P addition treatments. This observation showed that the larger the individual ramet (*i.e.,* number of buds), the greater the reserve growth potential, vegetative growth intensity, and sexual reproduction effort (Fig. 2). This is inconsistent with previous studies on dominant bud traits, which reported that apical dominance tended to decrease with increase in plant size (Bonser & Aarssen, 2003; Poorter et al., 2012). The reason for the difference might be that the relationship between meristem allocation and resource allocation is species dependent (Lehtilä & Sundås Larsson, 2005). However, we speculate that it may be associated with plant strategies to maximize adaptation across a range of environments (Kumar, Memo & Mastinu, 2020), such as fertilization treatment.

Previous studies (Ott & Hartnett, 2011; Baker et al., 2014) have focused on the allocation of new developmental meristems, which indicated the influence of apical dominance on the inactive meristem. In the present study, we investigated the dynamic changes of all buds of an individual plant, because it is difficult to distinguish and quantitatively study the new dormant buds from the older buds for woody plants. The allometry studied in this paper is only for the flowering stage, not the final developmental stage in the one growing season or whole life history. As a polycarpic perennial species, the reproductive buds of *N. tangutorum* in this paper refer to the inflorescence meristems, which have continuous growth similar to vegetative meristems after the reproductive stage. Essential for meristem allocation is that floral meristems are terminal (Lehtilä & Sundås Larsson, 2005). Thus bud dynamic pattern studied in the paper maybe not apply to the principle of allocation.

Perennialism requires, by definition, the maintenance of at least one indeterminate meristem that continues vegetative growth in the following season (Rohde & Bhalerao, 2007; Lundgren & Des Marais, 2020). Interannual variation in reproductive and vegetative growth may reflect adaptations to increase long-term survival or may be the result of environmental limitations on plant size and/or reproduction (Guo et al., 2012). Thus, describing and evaluating interannual variation in allometric growth of perennials enables interpretation of the underlying mechanisms of plant plasticity over multiple years in terms of growth processes, reproduction strategies, and environmental changes (Nakahata et al., 2021). In the present study, the allometry of the bud dynamic pattern of *N. tangutorum* showed significant differences among three consecutive years, which showed that the bud dynamic process participates in the cyclical process of growth–dormancy–growth for woody plants (Kotov & Kotova, 2010). Furthermore, much of the data that has been considered evidence for plasticity in reproductive allometry is actually evidence for plasticity in the rate of growth and development (Weiner et al., 2009). The detection of a correlation between two traits implicating an allocation tradeoff is dependent on low variation in the amount of resource acquired by the individuals. For perennial plants exposed to certain types of stress, the different types of meristematic buds could be stored to enable adaptation to resource availability in a different year (Ott, Klimesova & Hartnett, 2019). Such patterns of bud production in perennial grasses are important because the natality, longevity, and dynamics of buds determine the size of the dormant bud bank (Ott & Hartnett, 2011). For the above reasons, the bud dynamic pattern of *N. tangutorum*, as a clonal woody plant, differed significantly in different years, thereby providing a strategy for survival and reproduction of *N. tangutorum* in arid desert areas in the context of increasing deposition of N and P elements.

A large number of studies have shown that plant growth is strongly associated with the ecological stoichiometric characteristics (Elser et al., 2003; Pang et al., 2020). This correlation may indicate the type of nutrient restrictions on the plant (Elser et al., 2010). Generally, the C:N and C:P ratios of plants are indicative of the nutrient utilization efficiency (Li et al., 2017; Chen & Chen, 2021) and the state of the plant community structure, function, and productivity (Aanderud et al., 2018). The N:P ratio is not only characterized by plant nutrient limitation, which provides suggestions for vegetation management (Zeng et al., 2016), but also is a good indicator of the transformation between N and P limitation (Schreeg et al., 2014). For desert ecosystems, many physiological processes associated with desert plant N and P cycling may be constrained, such as N-fixation and nutrient mineralization (Huang et al., 2018). Thus, desert plants can survive in an environment with limited resources (*e.g.*, nutrients) only through rationally allocation the limited resources among meristems and/or organs (Sardans et al., 2017).

In the present study, the bud traits of *N. tangutorum* (except for log(*D* + *R*)) were significantly correlated with P content and P-related ratios, which revealed that the bud dynamics of *N. tangutorum* were limited by P more than by N. It might be that the meristematic capacity of the plant was expanded because of the increase in N uptake and accumulation, providing material for vegetative and reproductive growth when the plant is not limited by N availability. In our present field experiment, the same minimal amount of N was added in each P addition treatment to lessen differences in soil nutrient concentrations among the quadrats. Plant growth in terrestrial ecosystems is often limited by the availability of N or P. Liebig’s law of the minimum states that the nutrient in least supply relative to the plant’s requirement will limit the plant’s growth (Ågren, Wetterstedt & Billberger, 2012). In the present study, we measured the bud dynamics of *N. tangutorum* during the flowering stage each year. P element is strongly associated with the flowering phase of plant growth, and thus is a vital nutrient involved in stimulating and enhancing bud development and set, seed formation, and blooming (Ye et al., 2019). The present results also confirmed that the plant tissue nutrient contents and stoichiometric ratios are of theoretical and practical importance for understanding changes in bud development of woody plants.

## Conclusions

We investigated the dynamic pattern for aboveground buds and C:N:P stoichiometry of *N. tangutorum* ramets following N and P fertilizer addition in the natural nebkhas in the Ulan Buh Desert during three consecutive years. Fertilizer addition affects the reserve growth potential, vegetative growth intensity, and sexual reproduction effort of *N. tangutorum* ramets, which also fluctuate between years. The allometry of three bud pattern traits shows size dependence, *i.e.,* the larger the individual ramet, the greater the reserve growth potential, vegetative growth intensity, and sexual reproduction effort. The plant tissue P content, and the C:P and N:P ratios are strongly correlated with the bud traits, whereas the C and N contents, and C:N are not correlated with the bud traits.

##  Supplemental Information

10.7717/peerj.14934/supp-1 Supplemental Information 1 Raw data of buds number of *N. tangutorum* ramet

10.7717/peerj.14934/supp-2 Supplemental Information 2 Data of Carbon, Nitrogen and Phosphorus content of *N. tangutorum* ramet

## Additional Information and Declarations

### Data Availability

The following information was supplied regarding data availability: The raw measurements are available in the Supplemental Files.
